# Combining precision medicine and prophylaxis in oesophageal squamous cell carcinoma

**DOI:** 10.1038/s41416-020-01057-3

**Published:** 2020-09-22

**Authors:** Mark A. Baxter, Lindsay C. Spender, Russell D. Petty

**Affiliations:** 1 Medical Oncology, Division of Molecular and Clinical Medicine, Ninewells Hospital and Medical School, University of Dundee, Dundee, Scotland UK; 2 Tayside Cancer Centre, Ninewells Hospital and Medical School, NHS Tayside, Dundee, Scotland UK

**Keywords:** Oesophageal cancer, Oesophageal cancer

## Abstract

A trial update confirms improved survival for prophylactic elective nodal irradiation and addition of erlotinib to definitive chemoradiotherapy in oesophageal squamous cell carcinoma (ESCC). High tumour EGFR protein expression shows promise to identify those who will benefit from erlotinib. This represents therapeutic progress, and has wider relevance for precision medicine strategies in ESCC.

## Main

Oesophageal cancer comprises two main histological subtypes, oesophageal adenocarcinoma (OAC) and oesophageal squamous cell carcinoma (ESCC). ESCC is the most common subtype globally, and aetiological factors include tobacco, alcohol, specific dietary practices, and social deprivation.^1^ ESCC is an aggressive disease with 5-year overall survival (OS) of ~15%.^1^ The extensive oesophageal submucosal lymphatic plexus facilitates early dissemination of ESCC to regional lymph nodes and the majority of patients have micrometastatic or disseminated disease at the time of initial presentation.^1^

Definitive concurrent chemoradiotherapy (dCRT) is one standard of care for ESCC patients with loco-regionally confined disease with reported survival comparable to oesophagectomy and lymphadenectomy in randomised studies.^1^ However, loco-regional tumour persistence or recurrence, which is difficult to palliate, occurs in the majority and distant metastases in about half of dCRT-treated patients.^1–4^ To improve dCRT outcomes, approaches that combine better loco-regional tumour control with more effective systemic treatment of micrometastatic disease are required.

In this issue of the *British Journal of Cancer*, Xie et al.^5^ report a Phase 3 randomised controlled trial (RCT) with a 2 × 2 factorial design investigating whether prophylactic elective nodal irradiation (ENI) dCRT is superior to conventional field irradiation (CFI) and whether dCRT plus the epidermal growth factor receptor (EGFR) tyrosine kinase inhibitor (TKI) erlotinib, is superior to dCRT alone. ENI includes the uninvolved lymph nodes in the treatment field, which are at risk for micrometastatic disease, while CFI includes only the metastatic nodes. Several retrospective studies of dCRT with ENI in ESCC have reported reduced loco-regional failure, but some concerns remain regarding increased toxicity. EGFR has an established role in ESCC pathogenesis and EGFR inhibitors can reverse the radioresistance of oesophageal cancer cells. In this article,^5^ the authors provide a follow-up report on long-term outcomes, and importantly the results of a predictive biomarker analysis. They show that the early outcome benefits for ENI and erlotinib have been sustained. Key findings are that ENI improves OS compared to CFI (median OS, 38.5 vs. 22.6 months; hazard ratio [HR], 0.74; *P* = 0.018), and the addition of erlotinib also improves OS (median OS, 39.4 vs. 27.4 months; HR, 0.75; *P* = 0.025). The reported 5-year survival of 19.6% for CFI dCRT without erlotinib is entirely consistent with expectations, while 5-year OS of 44.9% for prophylactic ENI dCRT plus erlotinib represents an impressive incremental gain. Importantly, Xie et al.^5^ also provide reassurance regarding the late toxicity of ENI (plus erlotinib).

This is the first large RCT comparing ENI and CFI in ESCC and provides valuable data in this regard. The addition of anti-EGFR treatments to dCRT with CFI has been evaluated in previous RCTs, which demonstrated no benefit.^2–4^ The results presented here contrast with those from these earlier studies, which enrolled both OAC and ESCC and involved evaluation of the EGFR monoclonal antibody, cetuximab. In SCOPE-1, the addition of cetuximab to dCRT resulted in worse OS.^2^ In SAKK75/08, and RTOG 0436, OS was not improved with cetuximab, but in SAKK75/08, the addition of cetuximab did improve loco-regional control.^3,4^ Similarly, in biomarker unselected advanced ESCC patients, panitumumab added to cisplatin plus fluorouracil did not improve OS.^6^ Based on the findings of these studies and others, some consider that, despite strong evidence of its pathogenic role, EGFR is not a useful target for ESCC and OAC. However, this ignores consistent observations that, in biomarker-selected patients, anti-EGFR therapies in ESCC and OAC demonstrate efficacy.^7,8^ These studies demonstrate the predictive impact of *EGFR* gene copy number gain (CNG), or high EGFR protein expression.^7,8^ The new findings presented by Xie et al.^5^ extend this important observation, demonstrating that only patients with high EGFR expression (immunohistochemical [IHC] score +2, +3) appear to benefit from the addition of erlotinib to dCRT (median OS in erlotinib treated for EGFR IHC +2/3 vs. 0/+1, is 46.5 vs. 9.5 months; *P* = 0.007). The magnitude of benefit is striking. Taken together, these trials suggest therapeutic value for anti-EGFR treatments in ESCC that are EGFR IHC and/or *EGFR* CNG positive. However, even with appropriate biomarker selection, clinical resistance to anti-EGFR monotherapy in advanced stage ESCC (and OAC) often emerges rapidly,^7,8^ which suggests that combination treatments with EGFR inhibitors are likely to be important for meaningful clinical benefit—in this context, the positive findings by Xie et al.^5^ with erlotinib added to dCRT take on greater significance.

Recent positive findings for immune checkpoint inhibitors (ICIs) in advanced ESCC have understandably focussed attention on targeting PD-L1/PD-1.^9^ However, only a minority subgroup of patients respond to ICIs and in squamous cell carcinomas (SCCs), including ESCC, EGFR activation is associated with depleted tumour-infiltrating lymphocytes, resistance to ICI, and possibly hyper-progression.^10^ This suggests that precise targeting of EGFR-driven ESCC should remain an important therapeutic aim.

**Fig. 1:**
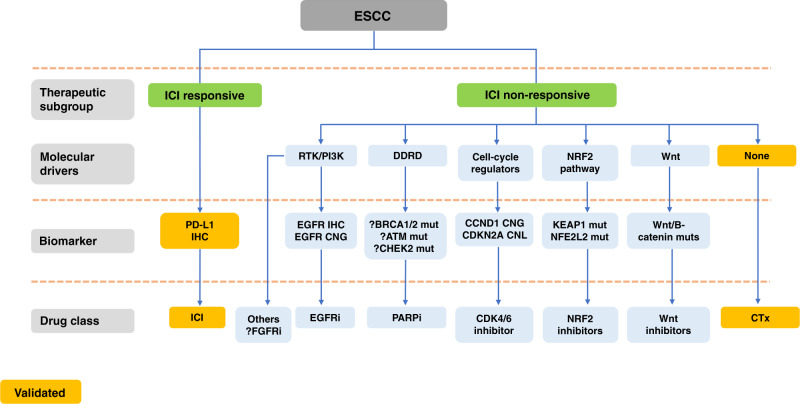
Emerging strategies and targets for ESCC precision medicine. IHC immunohistochemistry, ICI immune checkpoint inhibitor, PD-L1 programmed death-ligand 1, RTK receptor tyrosine kinase, DDRD DNA damage response deficient, CTx chemotherapy, CNG copy number gain, CNL copy number loss, EGFR epidermal growth factor receptor, PI3K phosphoinositide 3-kinase, mut mutant, *NRF2* nuclear factor, erythroid 2 like 2, CDK cyclin-dependent kinase, PARPi poly(ADR-ribose) polymerase inhibitor, FGFRi fibroblast growth factor receptor inhibitor, KEAP1 Kelch like ECH associated protein 1.

A key strength of the Xie et al.^5^ study is that it enrolled only ESCC. This is important in context of results of genomic landscaping studies, which indicate that ESCC has more similarity to SCC from other sites, than it does to OAC.^11^ Common genomic aberrations can be seen in SCCs when analysed at a pathway level, and targeting EGFR is perhaps best viewed in this context of emerging precision medicine targets and strategies for ESCC (Fig. 1).

A key limitation of this study^5^ includes the assessment of EGFR in only a minority subset, albeit one that was representative of the whole trial cohort. Prospective validation of EGFR IHC and/or *EGFR* CNG as a predictive biomarker is therefore needed. Also, the radiation dose of 60 Gy used is not widely practiced outside China and Japan, which will be a concern for many radiation oncologists, and makes the case for additional RCTs in western and other populations with more typically used radical radiation doses of 50–50.4 Gy.

Overall, the authors should be congratulated on completing a study that has efficiently addressed two important clinical questions for ESCC, and provided a signal that the combination of a precision medicine (erlotinib in EGFR IHC high) and prophylaxis (ENI) can provide very meaningful incremental survival benefit in ESCC. Considering the high unmet clinical need and the paucity of targets for precision treatments in ESCC, those such as EGFR for which we do see signals should be investigated comprehensively.

## Data Availability

Not applicable.
